# Modelling recurrent events: comparison of statistical models with continuous and discontinuous risk intervals on recurrent malaria episodes data

**DOI:** 10.1186/1475-2875-13-293

**Published:** 2014-07-29

**Authors:** Issaka Sagara, Roch Giorgi, Ogobara K Doumbo, Renaud Piarroux, Jean Gaudart

**Affiliations:** 1 Malaria Research and Training Center, Department of Epidemiology of Parasitic Diseases, Faculty of Medicine and Odonto-Stomatogy, University of Sciences, Techniques and Technologies of Bamako, BP 1805 Point G, Bamako, Mali; 2 Aix-Marseille University, UMR912 SESSTIM (INSERM, IRD, AMU), Marseille 13005, France; 3 Aix-Marseille University, UMR MD3, Marseille 13005, France

**Keywords:** Recurrent events, Malaria, Discontinuous risk intervals, Extended Cox model, Shared frailty model, GEE

## Abstract

**Background:**

Recurrent events data analysis is common in biomedicine. Literature review indicates that most statistical models used for such data are often based on time to the first event or consider events within a subject as independent. Even when taking into account the non-independence of recurrent events within subjects, data analyses are mostly done with continuous risk interval models, which may not be appropriate for treatments with sustained effects (e.g., drug treatments of malaria patients). Furthermore, results can be biased in cases of a confounding factor implying different risk exposure, e.g. in malaria transmission: if subjects are located at zones showing different environmental factors implying different risk exposures.

**Methods:**

This work aimed to compare four different approaches by analysing recurrent malaria episodes from a clinical trial assessing the effectiveness of three malaria treatments [artesunate + amodiaquine (AS + AQ), artesunate + sulphadoxine-pyrimethamine (AS + SP) or artemether-lumefantrine (AL)], with continuous and discontinuous risk intervals: Andersen-Gill counting process (AG-CP), Prentice-Williams-Peterson counting process (PWP-CP), a shared gamma frailty model, and Generalized Estimating Equations model (GEE) using Poisson distribution. Simulations were also made to analyse the impact of the addition of a confounding factor on malaria recurrent episodes.

**Results:**

Using the discontinuous interval analysis, AG-CP and Shared gamma frailty models provided similar estimations of treatment effect on malaria recurrent episodes when adjusted on age category. The patients had significant decreased risk of recurrent malaria episodes when treated with AS + AQ or AS + SP arms compared to AL arm; Relative Risks were: 0.75 (95% CI (Confidence Interval): 0.62-0.89), 0.74 (95% CI: 0.62-0.88) respectively for AG-CP model and 0.76 (95% CI: 0.64-0.89), 0.74 (95% CI: 0.62-0.87) for the Shared gamma frailty model.

With both discontinuous and continuous risk intervals analysis, GEE Poisson distribution models failed to detect the effect of AS + AQ arm compared to AL arm when adjusted for age category. The discontinuous risk interval analysis was found to be the more appropriate approach.

**Conclusion:**

Repeated event in infectious diseases such as malaria can be analysed with appropriate existing models that account for the correlation between multiple events within subjects with common statistical software packages, after properly setting up the data structures.

## Background

Recurrent events data analysis is quite common in biomedicine, such as low back pain, sick leave from work, sporting injuries, hospital readmissions and episodes of infectious diseases such as malaria [1-7]. Literature review indicates that most statistical models applied to such data are often based on naive techniques. Such naive techniques are characterized by either ignoring the existence of recurrent events, or ignoring the fact that the recurrent events within subjects are correlated [1,2]. Even when taking into account the non-independence of recurrent events within subjects, data analyses are mostly done with continuous risk interval models [2-5], which may not be relevant for health conditions with discontinuous risk [8]. In the medical field, it is quite common to encounter recurrent health conditions with such discontinuous risk intervals, e.g. in cases with persistent treatment effect. Examples include infections, such as malaria, disability episodes, hospitalizations, and nursing home admissions [7-12]. When subjects have a disability episode, they are not at risk of the second episode of disability until they have recovered from the first episode. To obtain unbiased estimates of incidence rates, the person-time period when the subject is not at risk should be excluded from the risk set. When analysing recurrent time-to-event outcomes with discontinuous risk intervals the subject is not at risk of another event while a previous one is ongoing or if the subject is under treatment. Appropriate models for analysing recurrent events data include marginal models or frailty or random coefficient analysis models [8-13], which take into account the non independence assumption of events within the subject.

Furthermore, in the case of malaria treatment trials, investigators assume that randomization is sufficient for controlling differential risk. However, the location of each subject is important as the risk exposure shows high spatial variations due to different environmental factors [14,15] that must be taken into account.

This work aimed at comparing different approaches analysing recurrent malaria episodes, with continuous and discontinuous risk interval models, in order to contribute identifying useful models to analyse such malaria data.

## Methods

### Study design

Data were collected from July 2005 to July 2007 in Bougoula-Hameau, Sikasso, in the south region of Mali. Patients were randomized as they came to the health centre from July 2005 to October 2006 (accrual period) to one of the artemisinin-based combination therapy (ACT) arm: artesunate + amodiaquine (AS + AQ) or artesunate + sulphadoxine-pyrimethamine (AS + SP) or artemether-lumefantrine (AL). The study’s main objective was to compare malaria incidence between these three ACT. Patients received the same initial treatment at each subsequent episode of uncomplicated malaria during the course of the study. The clinical data and results related to the main objective were published elsewhere [7].

### Statistical models and data analysis

Four models were used for the analysis of recurrent time-to-event outcomes: i) Generalized estimating equations (GEE) model using a Poisson distribution; and three extended Cox models: ii) the Andersen-Gill counting process (AG-CP), iii) the Prentice-Williams-Peterson counting process (PWP-CP); and iv) the Shared gamma frailty model. To take into account the recurrent structure of the data, models i) to iii) are marginal and model iv) uses a shared frailty term.

Both continuous and discontinuous time interval approaches were used with the four different models to analyse the data. A 14 day washout period was estimated after each episode treatment based on the pharmacokinetics of the study drugs. Model results were evaluated by comparing the risk ratio (RR) estimates and their standard errors (SE).

To analyse the impact of a confounding factor on results, a simulation of a risk exposure was done with two levels (high risk exposure *versus* low risk exposure) using a Binomial distribution with parameter (probability of being in the high risk exposure class) depending on the number of malaria episodes: 0.99 if the subject experienced >6 malaria episodes else 0.75 if the subject experienced > 4 malaria episodes, else 0.25 if subject experienced >1 episode and otherwise 0.01. This binary factor simulates, for example, two zones of different exposure. For analysing the impact of such a confounding factor, 1,000 independent replicates were performed, using R2.15.2 software (The R Foundation for Statistical Computing, Vienna, Austria). With the simulation data, only discontinuous time interval analysis was done with each of the four models. The impact of the simulated confounding factor on the results for each model was assessed by comparing the magnitude and confidence intervals of RR estimates, power and empirical coverage rates (ECR). For each covariate, the ratio between the standard error (SE) of the estimates using simulated data and the SE of the estimates using the observed data was computed. These criteria can be interpreted as the impact of the confounding factor on the estimation accuracy.

### GEE model using Poisson distribution

The Poisson regression model is frequently used to analyse count data or to study disease incidence and mortality when the dependent variable represents the number of independent events that occur during a fixed period of time [16]. The conditional mean of *Y* (number of events) can be written as:

(1) $$\mathrm{Ln}\left(Y|X,\beta \right)=X_{i}\beta$$

Where, *X*_*i*_*β* = *β*_0_ + *β*_1_*X*_1_ + *β*_2_*X*_2_ + … + *β*_*n*_*X*_*n*_; *Ln* is the natural logarithm (the canonical link between the linear predictors and the conditional mean of *Y*).

The GEE Poisson estimates the same model as the standard Poisson regression allowing for dependence within clusters. Therefore, it is appropriate to model recurrent events within a subject, such as in longitudinal data. The regression coefficients are refit, correcting iteratively for the correlation. In such models, the within-subject correlation structure is treated as a nuisance parameter. In this work the exchangeable correlation structure has been used assuming that the correlation between events remained constant through the time [17].

### Extended Cox models

The extended Cox models are used to model recurrent events within a subject unlike the Cox model, which is used to model a unique event or, sometimes, the first event. The considered Cox extended models were: the counting process model (Anderson-Gill model or AG-CP) [18], and the conditional model (Prentice-Williams-Peterson counting process model or PWP-CP) [16].

The sandwich robust standard error of Lin and Wei [3,19], which is a variance-correction technique, is usually employed together with these Cox extended models to avoid inflation of type I error due to multiple observations per individual which do not require specification of the correlation matrix.

### The Anderson-Gill model (AG-CP)

The formula is written as:

(2) $$\lambda_{\mathrm{ik}}\left(t/X,\beta \right)=〛{}_{\mathrm{ik}}\left(t\right)\lambda_{0}\left(t\right)e^{X_{\mathrm{ik}}\beta }$$

*λ*_*ik*_*(t*) represents the hazard function for the *k*^*th*^ event of the *i*^*th*^ subject at time *t*; λ_0_*(t)* represents the common baseline hazard for all events over time; *X*_*ik*_ represents the vector of *p* covariates processes for the *i*^*th*^ individual; *β* is a fixed vector of *p* coefficients; _*ik*_ is a predictable process, taking values in {1,0} indicating when the *i*^*th*^ individual is under observation.

The AG-CP model uses this counting process time-scale for all episodes. The time-scale does not reset to 0 after an episode (Table 1). Data for each subject needs to be entered in the counting process style, with a start time, stop time and censoring indicator for each event.

**Table 1:** Data structures for modelling recurrent time-to-event outcomes

**ID**	**Start**	**End**	**Episode**	**Order**	**Time**	**Treatment**	**Age (Years)**	**Quarter**
1	0	28	1	1	28	AS + SP	3.93	1
1	42	52	1	2	10	AS-SP	3.93	1
1	476	700	0	10	224	AS + SP	3.93	1
2	0	77	1	1	77	AS + AQ	1.15	1
2	91	375	1	2	284	AS + AQ	1.15	1
2	417	700	0	4	283	AS + AQ	1.15	1
3	0	28	1	1	28	AL	1.48	1
3	42	78	1	2	36	AL	1.48	1
3	150	700	0	5	550	AL	1.48	1

Data dictionary:

ID: study subject identification number; start: the start time of the interval (in days); end: the time (in days) at which the event occurs or the time of censoring; episode, the occurrence of malaria episode (yes = 1, no = 0); order: the order of the episodes, which is used only for the PWP-CP model; time: the number of days at risk that is calculated from subtracting end from start variables; treatment: the same malaria treatment given to the patient during each episode; Age (Years): the patients age at enrolment in years; quarter: the resident place or bloc of the patient in the village (old quarter = 1, new quarter = 0).

### Conditional model (Prentice-Williams-Peterson counting process-PWP-CP)

The PWP-CP model is similar to the AG-CP model but stratified by events. The formula is written as:

(3) $$\lambda_{\mathrm{ik}}\left(t|X,\beta \right)=〛{}_{\mathrm{ik}}\left(t\right)\lambda_{0k}\left(t\right)e^{X_{\mathrm{ik}}\beta }$$

*λ*_*0k*_*(t*) represents the event-specific baseline hazard for the *k*^*th*^ event over time. In this model, a subject is assumed not to be at risk for a subsequent event until a current event has terminated.

### The shared frailty model

The frailty model, introduced in the biostatistical literature by Vaupel *et al.*[20], and discussed in detail by Hougaard, Duchateau and Janssen, and Wienke *et al.*[21-23], accounts for the heterogeneity in baseline. This model is an extension of the proportional hazards model in which the hazard function depends upon an unobservable random variable. Subjects may be exposed to different risk levels, even after controlling for known risk factors, because of some relevant unobserved covariates. The frailty parameter models these unknown covariates. In a shared frailty model, individuals in the same group share the same frailty value which generates dependence between those individuals who share frailties.

The shared frailty model can be written as follows:

(4) $$\lambda_{\mathrm{ik}}\left(t/X,\beta ,u\right)=u_{i}\lambda_{\mathrm{ik}}\left(t\right)=\lambda_{0}\left(t\right)e^{X_{\mathrm{ik}}\beta +u_{i}}$$

Where *λ*_*ik*_ is the conditional hazard function for the *k*^*th*^ subject from the *i*^*th*^ cluster (conditional on *u*_*i*_); *λ*_0_(*t*) is the baseline hazard; *β* is the fixed effects vector of dimension *p*; *X*_*ik*_ is the vector of covariates; *u*_*i*_ is the random effect for the *i*^*th*^ cluster. Subjects in the same cluster u share the same frailty factor [22]. It is a conditional hazard model, given the *u*_*i*_. The cluster may represent a family, for example, or as in this case a single subject for which multiple episodes are observed.

The distribution of *u* may be Gamma, Gaussian, or other distribution. The gamma distribution has been chosen because of its mathematical tractability and because it is widely used [22]. The one-parameter chosen gamma distribution is defined as:

(5) $$f_{w}\left(u\right)=\frac{v^{1/\theta -1}e\left(-u/\theta \right)}{\theta^{\frac{1}{\theta }}\Gamma \left(1/\theta \right)}$$

with *Γ* the gamma function. Note that *E(u) = 1* and *Var(u) = θ*. This gives the following interpretation: subject in a class *i* with *u*_*i*_ *> 1* are frail, meaning of higher risk while subject with *u*_*i*_*<1* are strong, meaning of lower risk. The parameter *θ* informs on the clusters or classes heterogeneity in the population.

As in the Cox model or its extensions, the baseline hazard function for the frailty model does not vary by event, but the coefficient estimates of covariates effect from the frailty model, unlike the Cox model may vary if there is a significant random effect.

For the baseline hazard function, although other distribution could be used, the Weibull proportional hazards distribution was assumed. Weibull distributed event times are often used in practice, because they are able to describe the actual evolution of the hazard function in an appropriate way in many circumstances. Furthermore it is a popular flexible parametric model that allows the inclusion of covariates of the survival times [22].

### Data structure

The duration of each subject in the study was defined as the time between enrolment and the end of the study or until the subject is lost to follow or withdrawn. A malaria episode (event) had to be preceded and followed by a time period without malaria except in the case of withdrawal and at the end of the study period. As an example, Table 1 provides data for three study subjects (one in each study arm). Subject 1 had nine malaria episodes at days 28, 52, 80, 109, 305, 326, 410, 438 and 462 and the follow-up ended on day 700. Subject 2 had three malaria episodes at days 77, 375 and 403 and the follow-up ended on day 700. Subject 3 had four malaria episodes at days 28, 78, 105 and 136 and the follow-up ended on day 700. The duration of each malaria episode is 14 days as it is assumed that the subject is not at risk of new infection for this duration after treatment initiation (discontinuous risk interval data structure).

The data are organized as one record per subject per event. The data structure for the AG-CP model consists of the first four columns. A subject with multiple events is considered as multiple subjects for analytic purposes. For example, subject 2 is considered four times: the first begins follow-up at time 0 and has an event at 77 days; according to the fact that the subject is not at risk during 14 days, the second has delayed entry at 91 days and has an event at 375 days; the third has delayed entry at 389 days and has an event at 403 and is followed through 700 days without having an event. Because the counting process model does not consider the order of the events, it does not use the “order” column. In the PWP-CP model, a subject is assumed not to be at risk for a subsequent event until the current event has terminated. This means, one cannot be at risk for the second event without having experienced and completed the first event. The data structure for PWP-CP model is similar to that of the counting process AG-CP model except that the “order” column is also used to identify the event order. An Additional file 1 shows statistical codes for each model using Stata and R Software [see Additional file 1].

### Incidence rate and relative risk estimate of recurrent events

The incidence rate was computed as the number of events per person-days [24] and can be calculated from Table 1 as follows:

$$\frac{\sum_{k=1}^{n}\mathrm{even}t_{\mathrm{ij}}}{\sum_{k=1}^{n}\mathrm{tim}e_{\mathrm{ij}}}$$

where *event*_*ij*_ is the event status (1 or 0) for the *i*^*th*^ subject in the *j*^*th*^ interval; *time*_*ij*_ is the time at risk for the *i*^*th*^ subject in the *j*^*th*^ interval; *n* is the number of subjects. The relative risks (RRs) were computed to assess the treatment effect (AL study arm was used as the reference treatment) and the age group effect (age group < 5 years old, 5-9 years old and >9 years old. The age group >9 years old was used as reference group) on the occurrence of malaria episode. The hazard ratio (HR) for extended Cox models and the relative risk (RR) for the GEE model were estimated.

### Ethical considerations

The study has been approved from the institutional ethical committee (FWA #00001769) at the Faculty of Medicine, Pharmacy and Odonto-Stomatogy (FMPOS)/USTTB, Bamako, Mali.

A written consent was also obtained from each participant or their parent/legal guardian.

## Results

From July 2005 to July 2007, the 777 subjects enrolled into the study yielded a total of 1,649 malaria episodes (min = 1, max = 12, median = 2 episodes per subject). Using the discontinuous risk interval analysis, PWP-CP, AG-CP, and the Shared gamma frailty models provided larger treatment effect on malaria episodes compared to GEE for the patients treated with AS + AQ or AS + SP as compared to the AL arm; RRs were: 0.75 (95% CI (Confidence Interval): 0.62-0.89), 0.74 (95% CI: 0.62-0.88) respectively for AG-CP model, 0.76 (95% CI: 0.64-0.89), 0.74 (95% CI: 0.62-0.87) for the Shared gamma frailty model and 1.02 (0.93-1.11), 0.93 (0.87-0.99) for GEE model (Table 2). Similarly for the age category, using the discontinuous (Table 2) risk interval analysis, PWP-CP, AG-CP and the Shared gamma frailty models provided similar and higher magnitude of RRs for the patients in age group <5 years old or age group between 5-9 years old compared to patients of age group >9 years old; RRs were: 3.16 (95% CI: 2.15-4.65), 2.61 (95% CI: 1.76-3.88) respectively for AG-CP model and 3.04 (95% CI: 2.27-4.09), 2.54 (95% CI: 1.87-3.45) respectively for Shared gamma frailty model. The effect of covariates (treatments and age category) on malaria episodes were slightly higher for both AG-CP and the Shared gamma frailty models in discontinuous risk intervals (Table 2) compared to continuous risk intervals (Table 3).

**Table 2:** Coefficient estimates according to model by discontinuous risk intervals analysis

**Models**	AS + AQ* RR/HR (SE); [95% CI]; *p*	AS + SP* RR/HR (SE); [95% CI]; *p*	<5 years** RR/HR (SE); [95% CI]; *p*	5-9 years** RR/HR (SE); [95% CI]; *p*
GEE, Poisson distribution	1.02 (0.044); [0.93-1.11]; p = 0.722	0.93 (0.029); [0.87- 0.99]; p = 0.018	1.36 (0.201); [1.02-1.82]; p = 0.036	1.22 (0.181); [0.91-1.63]; p = 0.175
AG-CP	0.75 (0.068); [0.62-0.89]; p < 0.001	0.74 (0.065); [0.62-0.88]; p < 0.001	3.16 (0.621); [2.15-4.65]; p < 0.001	2.61 (0.526); [1.76-3.88]; p < 0.001
PWP-CP	0.86 (0.055); [0.76-0.97]; p = 0.015	0.85 (0.052); [0.75-0.96]; p = 0.007	2.34 (0.389); [1.69-3.24]; p < 0.001	2.04 (0.345); [1.46-2.84]; p < 0.001
Shared gamma frailty	0.76 (0.064); [0.64-0.89]; p = 0.001	0.74 (0.063); [0.62-0.87]; p < 0.001	3.04 (0.458); [2.27-4.09]; p < 0.001	2.54 (0.397); [1.87-3.45]; p < 0.001

*Reference treatment: AL.

**Reference age group: >9 years old.

CI: Confidence interval for RR/HR (Relative risk/Hazard ratio); SE: Standard error; p: p value.

**Table 3:** Coefficient estimates according to model by continuous risk intervals analysis

**Models**	AS + AQ* RR/HR (SE); [95% CI]; *p*	AS + SP* RR/HR (SE); [95% CI]; *p*	<5 years** RR/HR (SE); [95% CI]; *p*	5-9 years** RR/HR (SE); [95% CI]; *p*
GEE, Poisson distribution	1.02(0.044); [0.93-1.11]; p = 0.722	0.93 (0.029); [0.87- 0.99]; p = 0.02	1.36 (0.201); [1.02-1.82]; p = 0.04	1.22 (0.181); [0.91-1.63]; p = 0.18
AG-CP	0.77 (0.064); [0.65-0.91]; p = 0.002	0.76 (0.062); [0.65-0.89]; p = 0.001	2.94 (0.559); [2.03-4.27]; p < 0.001	2.48 (0.481); [1.69-3.62]; p < 0.001
PWP-CP	0.83 (0.053); [0.73-0.94]; p = 0.004	0.81 (0.050); [0.72-0.92]; p = 0.001	2.35 (0.388); [1.71-3.26]; p < 0.001	2.05 (0.344); [1.48-2.85]; p < 0.001
Shared gamma frailty	0.77 (0.061); [0.66-0.90]; p = 0.001	0.76 (0.060); [0.65-0.89]; p < 0.001	2.88 (0.415); [2.17-3.82]; p < 0.001	2.43 (0.364); [1.82-3.26]; p < 0.001

*Reference treatment: AL.

**Reference age group: >9 years old.

CI: Confidence interval for RR/HR (Relative risk/Hazard ratio); SE: Standard error; p: p value.

Using both discontinuous (Table 2) and continuous (Table 3) risk intervals analysis, GEE Poisson models did not find a preventive efficacy for the patients treated with AS + AQ arm compared to AL arm. Furthermore, the GEE models estimated a protective efficacy (1-RR) of lower magnitude for AS + SP than the 3 other models. The discontinuous (Table 2) and continuous (Table 3) risk intervals analysis results were similar for GEE Poisson model.

**Figure 1:**
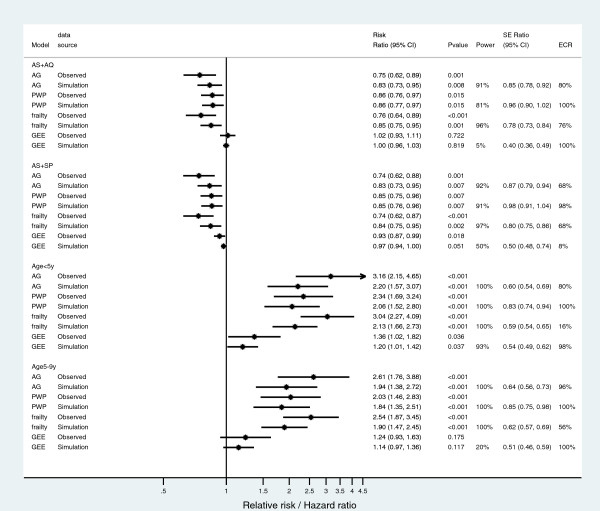
**Coefficients estimate using observed and simulated data using discontinuous risk intervals analysis.** Dark dots with lines are Relative risk/Hazard ratio and their 95% Confidence intervals respectively; Vertical central dark line is either the no effect treatment line compared to the referent category (artemether-lumefantrine) or the no effect age group compared the referent category (age group > 9 years old). Abbreviations: AS + AQ, artesunate + amodiaquine; AS + SP, artesunate + sulphadoxine-pyrimethamine; AG, Andersen-Gill; PWP, Prentice-Williams-Peterson; GEE, generalized estimating equation; SE, standard error; ECR, empirical coverage rate.

Incidence rates (Table 4) were slightly higher in the discontinuous interval analysis compared to the continuous interval analysis as the person-time was lower, although the 5% significance level was not reached for the incidence rate differences between treatment groups (exact mid p-values).Assessing the impact of risk exposure covariate with the simulated data (Figure 1), treatment effect estimates (AS + AQ and AS + SP compared to AL) were relatively lower than those with observed data for the AG-CP and the Shared gamma frailty models, but still remain significant for each extended Cox models. For the GEE model, there were no significant treatment at 5% for both AS + AQ and AS + SP compared to AL. Simulated data (Figure 1) confirmed the significant (at 5% significant level) treatment effect of AS + AQ and AS + SP compared to AL with power > 80% for all models except the GEE Poisson distribution model. Also, the age category effects (age category < 5 years and age category 5-9 years old compared to the age category > 9 years old) on malaria episodes were relatively lower compared to the effects of the age category on malaria episodes using the observed data analysis. For the GEE model, there were moderate to no significant age category effects.The simulated data (Figure 1) showed that adding a significant covariate reduces estimation variances. For the AS + AQ treatment, the standard error ratio (simulated over observed) for the AG-CP model was 0.72 (2.5-97.5 percentile [0.60-0.85]), while it was 0.16 (2.5-97.5 percentile [0.13-0.24]) for GEE Poisson distribution model. For the AS + SP treatment, the standard error ratio (simulated over observed) for the AG-CP model was 0.75 (2.5-97.5 percentile [0.63-0.89]), while it was 0.25 (2.5-97.5 percentile [0.23-0.54]) for GEE Poisson distribution model. The AS + SP effect estimates was clearly modified when using the GEE model (ECR = 7.7%), similarly to small age categories effect estimates when using the Shared gamma frailty model (ECR = 16.1%). AG-CP and PWP-CP models estimates were particularly stable after the addition of the simulated confounding covariate.

**Table 4:** Incidence rate* per treatment arm according to discontinuous or continuous risk intervals analysis

**Model**	**AL**	**AS + AQ**	**AS + SP**
	**IR [95% CI]**	**IR [95% CI]**	**IR [95% CI]**
Discontinuous time risk intervals	2.01 [1.86-2.17]	1.52 [1.39-1.66]	1.50 [1.37-1.64]
Continuous time risk intervals	1.87 [1.73-2.02]	1.44 [1.32-1.57]	1.42 [1.30-1.55]
*p-value*	0.09	0.19	0.19

*Malaria episodes/person/year.

IR: Incidence rate; CI: Confidence interval for IR; *p-values*: Exact mid p-values for risk difference between discontinuous and continuous risk intervals.

## Discussion

Methods are available to analyse data with recurrent events while accounting for the lack of independence among events [3-6,8-13]. In this paper, when focusing on recurrent malaria episodes data, results were different according to the model used, highlighting the importance of the model choice according to the medical question studied and the collected dataset. Two other survival models for recurrent events [3,8,20] have been proposed: Wei-Lin-Weissfeld (WLW), Prentice-Williams-Peterson gap time (PWP-GT). Various earlier studies have discussed the difficulty in conceptualizing the risk set of the WLW model and the biases in the estimates from the WLW and PWP-GT models [3,6,25], so these two models were not considered here. There is a limited applied statistical research modelling malaria recurrent episodes data [12], though malaria is one of the most devastating diseases in sub-Saharan Africa. The literature review indicates that, this is the first applied statistical research modelling using the main extended Cox models on recurrent malaria episodes data with both discontinuous and continuous risk intervals. The data structures have been efficiently prior organized with discontinuous risk intervals in order to perform the analysis using these different models. For these analyses, the time while the subject is not at risk because of the malaria treatment has to be taken into account and excluded from the risk set. There is a need of careful preparation of the data structure before applying theses analyses.

The AG-CP and the Shared gamma frailty models, with both discontinuous and continuous risk intervals provided similar parameter estimates of treatment effects (with respect to the referent treatment) or for age category effects (in respect to age referent category) on malaria recurrent episodes. This was previously observed by others [8] where the authors reported significant covariate effect estimates using AG-CP and the Shared gamma frailty models with discontinuous risk intervals analysis. AG-CP models are known to be useful and robust if one is interested in the overall effect, such as the treatment effect in a clinical trial, and if there is no clear biological mechanism underlying the relation between the first event and subsequent events [8,11]. The actual used data seems adapted to this described situation where having the first malaria episode does not preclude subsequent malaria episode as long as exposure is present (transmission condition), and is taken into account. The counting process (or AG-CP) model requires few assumptions, and is comparably robust like the traditional Cox regression model [3,18]. Although the Shared gamma frailty models generated similar results to the AG-CP model in this study, the specification of the frailty distribution may affect the coefficient estimates and more research is still needed in this area [8,22].

AS + SP, then followed by AS + AQ showed significant protective effects (1-RR) against recurrent malaria episodes compared to AL using the three extended Cox models (AG-CP, PWP-CP and the Shared gamma frailty). This could be explained by the longer half-life of the partner drugs (AQ and SP) and this observation has been reported previously [7]. In contrast, both the age category of <5 years old and the age category of 5-9 years old were significantly at higher risk of recurrent malaria episodes compared to old children and adults (>9 years old) using the three extended Cox models (AG-CP, PWP-CP and the Shared gamma frailty).

Time discontinuous or continuous risk intervals models should be chosen based on the disease or outcome conditions. In this case, the discontinuous risk intervals model should be chosen for unbiased estimates of covariate coefficients as shown in these data and also reported previously [8]. It is also more appropriate from an epidemiological point of view to take into account the time a subject is not at risk for a disease in a given period of time.

Less or no significant covariate effects (with respect to referent category) were found on malaria recurrent episodes using GEE for both discontinuous and continuous risk intervals models which yielded similar coefficient estimates. GEE is considered a very flexible approach [26]. Indeed, GEE models can handle a variety of correlated measure models that arise from recurrent episodes measure in the same individuals over time [27-29]. GEE models are robust to misspecification of the correlations structures [26]. Selecting robust standard errors (Huber/White Sandwich Estimators; as opposed to conventional standard errors) allow the estimates to be valid even if the correlation structure is misspecified. The poor performance of GEE, shown here, may also be explained by inappropriate assumption of the Poisson distribution [30] as the data maybe underdispersed with 70% of the dependant value count greater than zero.

Analyses including the simulated variables also showed significant treatments (AS + AQ and AS + SP using AL as referent category) and age category (using >9 years old as referent category) effects on malaria recurrent episodes with AG-CP, PWP-CP and the Shared gamma frailty models as in observed data analysis though these effects were more important for AG-CP and the Shared gamma frailty models.

The simulation study showed that a risk exposure factor modified the estimates, reducing the treatment effects. In malaria studies conducted in sub-Saharan countries, the main risk exposure factor includes the house location of subjects [14,15] due to specific environments, which influence malaria transmission. The distribution of mosquito breeding sites within villages is not uniform, thus, the exposure varies within villages, and this must be taken into account when assessing treatment effects. As shown in Figure 1, the simulation data provided proof of an existing confounding factor that needs to be taken into account. In fact, the RRs and their confidence intervals estimated on simulated data trended toward the null hypothesis (more close for RRs confidence intervals to include 1) compared to the RRs and their confidence intervals estimated on observed data. These variations were more important for the AG-CP and the Shared gamma frailty models despite their power (ability to detect significant effect) than in the PWP-CP and GEE Poisson distribution models. But, according to the empirical coverage rate as described by Burton *et al.*[31], the PWP-CP remained the more robust to the addition of the simulated confounding factor.

## Conclusions

In the context of malaria, statistical models have to take recurrent events and discontinuous time into account to better estimate effects of covariates such as treatments or risk factors such as age on malaria recurrent episodes. In this context, the three extended Cox models presented here are of high interest and showed similar results.

## Competing interests

The authors do not have any competing interests to declare.

## Authors’ contributions

IS conceived the study, prepared the data, carried out the data analysis, wrote an initial draft of the manuscript, and worked on the production of final draft. RG helped in the study, advised on data analysis, and contributed to the writing of the manuscript. OKD contributed to the interpretation of the results and the writing of the manuscript. RP contributed to the interpretation of the results and the writing of the manuscript. JG helped in the study conception, advised on the data preparation, contributed to the data analysis, and worked on the production of final draft. All authors read and approved the final manuscript.

## Supplementary Material

Additional file 1 Stata (Stata Corporation 2011) and R codes for the different models. Click here for file
