# Supplementary material for: Modelling recurrent events: comparison of statistical models with continuous and discontinuous risk intervals on recurrent malaria episodes data
Source: Malar J. 2014 Jul 29;13:293. doi: 10.1186/1475-2875-13-293 (PMC4132199; doi:10.1186/1475-2875-13-293)
Supplement: Additional file 1 — Stata (Stata Corporation 2011) and R codes for the different models. [file 1475-2875-13-293-S1.pdf]

Additional file 1 Stata (Stata Corporation 2011) and R codes for the different models

**Models**	**Stata codes**	**R codes**
**GEE Poisson distribution**	xi: xtgee episode i.treatcat i.agecat, eform f(poisson) i(ID) l(log) cor(exch) t(time) robust	mod_gee <- gee(episode~ treatcat + agecat, data = table_long, id = ID, family = poisson, corstr = "exchangeable")
**AG-CP**	xi: stcox i.treatcat i.agecat, efron nolog cluster(ID)	mod_AG<-coxph(Surv(start, end, episode)~treatcat+agecat+ cluster="ID", data= table_long)
**PWP-CP**	xi: stcox i.treatcat i.agecat, efron nolog cluster(ID) strata(order)	mod_PWP<- coxph(Surv(start, end, episode)~treatcat+agecat+ cluster="ID", strata="order",data= table_long)
**Frailty Gamma**	xi: streg i.treatcat i.agecat, d(weib) frailty(gamma) shared(ID) nolog	mod_frailty<- coxph(Surv(start, end, episode)~treatcat+agecat+ frailty (ID, dist="gamma"),data= table_long)

Variables names and signification are provided in the data dictionary in Table 1.

For Stata codes:

For GEE Poisson distribution: xi: meaning using dummy codes for independent variables (i.treatcat=treatment category, i.agecat=age. Dummy codes for treatment category were: AL=0, AS+AQ=1, AS+SP=2; Dummy codes for age category were: <5year=2, 5-9years=1, >9years=0; xtgee function fits a population-averaged panel-data models by using GEE; eform is the displaying function for the exponentiated coefficients; f(poisson) is poisson family function; i is the patient identification variable; l(log) is logarithm link function; cor(exch) is the correlation structure function with exchangeable option; t(time) is the time function linking the time/duration (days here); robust is a roust standard error function.

For AG-CP, PWP-CP and Frailty Gamma models, there is a need to declare the survival expression by the following syntax: stset end, failure(episode) exit(end) enter(start).

For R codes: require gee and survival packages.

For GEE Poisson distribution: mod_gee is the object name; gee is a function for generalized estimation equation; table_long is the data format used in Table 1; id is the patient identification variable; corstr is the correlation structure.

For AG-CP and PWP-CP models: mod_AG and mod_PWP are the object name respectively for AG and PWP-CP models; coxph is the Cox Proportional hazard function; Surv is the Survival function; strata fits a stratified Cox model by ordered malaria episodes.
For Frailty Gamma model: mod_frailty is the object name; frailty is the shared frailty function within patient with gamma distribution.
